# Endovascular Recanalization of Non-acute Symptomatic Middle Cerebral Artery Total Occlusion and Its Short-Term Outcomes

**DOI:** 10.3389/fneur.2019.00484

**Published:** 2019-05-15

**Authors:** Meimei Zheng, Yun Song, Jinping Zhang, Wei Zhao, Lili Sun, Hao Yin, Jun Zhang, Wei Wang, Ju Han

**Affiliations:** Department of Neurology, Shandong Provincial Qianfoshan Hospital, The First Hospital Affiliated with Shandong First Medical University, Shandong University, Jinan, China

**Keywords:** endovascular recanalization, angioplasty/stenting, total occlusion, middle cerebral artery, drug-coated balloon, outcome

## Abstract

**Background and Purpose:** The optimal treatment for patients with non-acute symptomatic middle cerebral artery (MCA) total occlusion and a high risk of recurrent ischemic stroke despite medical management is not well-established. We aimed to assess the feasibility, safety, and short-term outcomes of angioplasty and stenting for these patients.

**Methods:** Data of 22 patients with non-acute symptomatic MCA total occlusion who have failed medical management and undergone endovascular recanalization were retrospectively collected in our prospective database. All occlusive lesions were predilated with conventional balloons, followed by paclitaxel-coated coronary balloon inflation or not, and then a remedial stenting was performed or not, depending on the discretion of the operator. The rate of successful recanalization, perioperative outcomes, and short-term outcomes, such as restenosis and stroke recurrence, was analyzed.

**Results:** Successful recanalization was achieved in 95.5% of patients, with 14 patients undergoing balloon angioplasty and 7 patients undergoing remedial stenting. Seven patients developed perioperative complications, including one patient with persistent neurological deficit. Over a median clinical follow-up duration of 5.0 months, only one patient had recurrent ischemic symptoms attributed to the cessation of antiplatelet treatment owing to postoperative intracranial hemorrhage. The proportion of patients who achieved favorable clinical outcome (modified ranking scale score of 0–2) was 85.7%. Post-procedural repeat vascular imaging was performed at 4.5 ± 1.84 months, with nine and one patient undergoing cerebral angiography and magnetic resonance angiography, respectively. One (10%) artery presented with asymptomatic reocclusion.

**Conclusions:** Angioplasty and stenting may be feasible for the patients with non-acute symptomatic atherosclerotic MCA total occlusive disease who have failed medical management.

## Introduction

Intracranial arterial occlusion (IAO) or stenosis is one of the main causes of ischemic stroke in China. In the Chinese Intracranial Atherosclerosis (CICAS) Study, ~33% of ischemic stroke patients had large IAO, and among patients with intracranial atherosclerotic (ICAS) disease, recurrent stroke occurred in 7.27% of patients owing to occlusion and in only 5.16% of patients owing to 70–99% stenosis during 1-year clinical follow-up, and resulted in neurologic functional deterioration (1). Moreover, IAO may also lead to chronic cognitive impairment and poor quality of life (2). The CICAS study also revealed that the occlusive lesion in the middle cerebral artery (MCA) was found in 14.18% of patients (406/2,864), which ranked first among all the intracranial arteries (1).

A large number of patients with acute ischemic stroke attributed to MCA occlusion are unable to reach a qualified stroke center in time after symptom onset; thus, they did not undergo immediate thrombolytic therapy or mechanical thrombectomy. Moreover, the proportion of successful recanalization after intravenous thrombolytic therapy is only ~10%, with the majority of patients being left with an occluded MCA and still being at a higher risk of recurrent stroke. Patients with chronic MCA occlusion may have minor stroke or be asymptomatic because of well-developed collateral circulation between the anterior cerebral artery and posterior cerebral artery through leptomeningeal anastomosis. Conversely, the prognosis for those with hemodynamic impairment and cerebral misery perfusion is much poorer because of hemodynamic cerebral infarction and disabling cognitive impairment despite the best medical therapy (BMT) (3).

However, currently, no consensus exists on the optimal treatment of these non-acute MCA occlusions. The efficacy of extracranial–intracranial arterial bypass has yet to be conclusively demonstrated in large studies even for hemodynamic compromised patients (4). Percutaneous transluminal angioplasty and stenting (PTAS) or angioplasty alone may be a potential treatment option with the development of new endovascular devices including drug-coated balloon (DCB). Vessel recanalization appears to have a powerful effect on clinical outcomes in acute ischemic stroke with large vascular occlusions (5, 6). However, physicians are reluctant to treat subacute or chronic total MCA occlusions using angioplasty/stenting considering technical difficulties and perioperative complications. Only a few case reports have revealed that endovascular recanalization appeared to be a tool to prevent stroke recurrence (7–10).

In this single-center study, we aimed to assess the feasibility and the safety of angioplasty and PTAS and to investigate their short-term clinical and angiographic outcomes in patients with non-acute MCA occlusion.

## Materials and Methods

### Study Population

This was a retrospective study based on data obtained from our prospectively maintained database in Shandong Provincial Qianfoshan Hospital, Shandong University. From January 2015 to August 2018, we recruited consecutive patients diagnosed with symptomatic MCA occlusion by digital subtraction angiography (DSA) who failed medical management and underwent angioplasty or PTAS according to the following inclusion criteria: (1) intracranial atherosclerosis was the primary etiology; (2) hemodynamic failure in the MCA territory, which was supposedly based on the following clinical and imaging evidence: (a) recurrent transient ischemic attack (TIA), stroke, neurologic deterioration (progressive or crescendo stroke), unsatisfactory improvement of symptoms despite BMT (including dual-antiplatelet therapy, statin, blood pressure augmentation therapy, optimized glucose control, as well as smoking cessation and emphasis on healthy lifestyle), or severe ischemic symptoms not explained by a corresponding infarct (i.e., clinical infarct mismatch); (b) infarctions located at the watershed area or cortical/subcortical area on magnetic resonance (MR) imaging in the MCA territory; and (c) small infarction core shown on diffusion-weighted MR with a large area of low perfusion of MCA territory assessed by arterial spin labeling; and (3) DSA showed favorable patency of distal vasculature.

The exclusion criteria were as follows: (1) with other potential causes of occlusion, such as vasculitis, Moyamoya syndrome, or arterial dissection; (2) there was no recurrent ischemic events after BMT; and (3) collateral circulation between the anterior cerebral artery and/or posterior cerebral artery was well-developed, and perfusion of MCA territory assessed by arterial spin labeling was adequate.

All enrolled patients or their authorized surrogate gave written informed consent in accordance with the Declaration of Helsinki. This study was approved by the institutional review board of Shandong Provincial Qianfoshan Hospital, Shandong University. Given its retrospective nature, the study does not require registration.

### Intervention Procedure

All procedures were performed under general anesthesia. Intravenous heparin boluses were administered to maintain the activated clotting time between 250 and 300 s during the procedure. The vessel pathway was comprehensively assessed before the intervention. A Synchro micro-guidewire (Stryker Neurovascular, Utah, USA), assisted by an Excelsior SL-10 soft micro-catheter (Stryker Neurovascular, Cork, Ireland), was introduced into the occluded MCA and carefully passed through the occluded lesion to the distal segment. After withdrawing the micro-guidewire, the length of the occlusion and distal lumen of the lesion were confirmed through the micro-catheter angiography. Thereafter, the micro-guidewire reached the M3 segment, and the micro-catheter was withdrawn. The lesions were initially inflated with conventional balloons. The subsequent application of paclitaxel-coated coronary balloon (SeQuent Please, B. Braun, Berlin, Germany) and stent (Wingspan stent, Boston Scientific, Massachusetts, USA; Solitaire AB stent, Medtronic, Minneapolis, USA; Neuroform EZ stent, Boston Scientific, Massachusetts, USA; Enterprise stent, Cordis Neurovascular, Florida, USA) depended on the operator's decision. The application of DCB has been detailed in our previous study (10). Post-procedural antegrade flow was graded using the thrombolysis in cerebral ischemia (TICI) grading system (11).

### Pre-Procedural and Post-Procedural Medical Management

Before the procedure, the patients were administered aspirin (100 mg daily) and clopidogrel (75 mg daily) for 5 days, and thromboelastography platelet mapping was performed to guide the modulation of antiplatelet treatment. After the intervention, blood pressure was maintained at 100–120/60–80 mmHg. Patients were required to take aspirin 100 mg/day and clopidogrel 75 mg/day for 3 months for angioplasty and 6 months for stenting, and then continued with either one of the two drugs. Patients were also prescribed with statins and educated on the ways for controlling other risk factors.

### Follow-Up Management and Data Collection

After the intervention, cranial CT scan was performed to identify potential intracranial hemorrhage (ICH). All the successfully reopened patients were followed up clinically at 1, 3, and 6 months and were scheduled for repeat vascular imaging examination at 3 months (for angioplasty) or 6 months (for remedial stenting).

Demographic, clinical, procedural, and angiographic data were collected. The modified Rankin Scale (mRS) and the National Institutes of Health Stroke Scale (NIHSS) were applied at admission, pre-procedure, 24 h post-procedure, at discharge, 30-day post-procedure, and every follow-up. Technical success was determined by recanalization with TICI grade ≥2b. Complications investigated included MCA branch embolization, ICH, hyperperfusion syndrome, perforating branch occlusion, vessel perforation, dissection, and death. A favorable clinical outcome was defined as mRS score of 0–2. Restenosis and reocclusion were defined as a diameter of the stenosis >50% or total occlusion of the target artery segment, respectively. Symptomatic restenosis was defined as restenosis associated with ischemic symptoms of the offending vessel territory.

### Statistical Analysis

Continuous data are expressed as mean ± SD or as the median with interquartile range (IQR), whereas categorical data were presented as numbers and percentages. All statistical analyses were performed using SPSS software (SPSS Inc., Chicago, USA).

## Results

### Patients' Characteristics

Twenty-two patients with male predominance (17/22, 77.3%) were enrolled (Supplementary Table l). Baseline demographic and clinical characteristics of the 22 patients are listed in Table 1. Median mRS and NIHSS scores at baseline were 1.0 (1.0–4.0) and 2.5 (0.75–5.75), respectively.

**Table 1:** Patients' baseline demographic and clinical characteristics.

**Characteristics**	***n* = 22**
**Demographics**	
Sex, male	17 (77.3%)
Age, years, mean ± SD	57.09 ± 8.25
**Medical History**	
Hypertension	16 (72.7%)
Diabetes mellitus	10 (45.5%)
Dyslipidemia	9 (40.9%)
Cardiovascular disease	2 (9.1%)
Smoking Atrial fibrillation	11 (50.0%) 0 (0%)
**Clinical**	
mRS, median (IQR)	1.0 (1.0–4.0)
NIHSS, median (IQR)	2.5 (0.75–5.75)
Preoperative NIHSS, median (IQR) **Timing**	2.0 (0–7.0)
Symptom onset to treatment, days, median (IQR)	17.5 (14.75–29.25)
Occlusion confirmed to treatment, days, median (IQR)	12.5 (7.75–16.75)

*mRS, modified Rankin Scale; NIHSS, National Institutes of Health Stroke Scale; SD, standard deviation; IQR, interquartile range*.

### Procedural Characteristics

**Figure 1:**
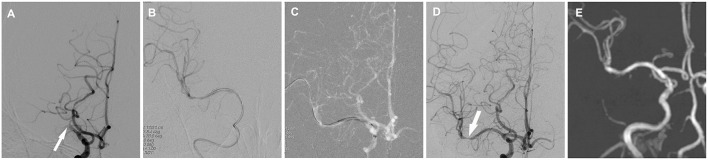
Cerebral angiographic results of drug-coated balloon (DCB) dilatation for patient 10 during the procedure and follow-up. **(A)** Total occlusion of the right middle cerebral artery (arrow). **(B)** Angiographic result after predilatation with a conventional balloon. **(C)** DCB dilatation after predilatation. **(D)** Angiographic result after the procedure with a residual stenosis (arrow) and good antegrade perfusion. **(E)** MR angiographic result at the 3.5-month follow-up with a patent middle cerebral artery.

Overall, the median time from symptom onset to treatment was 17.5 days, and the median time from radiographic documentation of occlusion to treatment was 12.5 days. As shown in Table 2, 14 patients were treated with angioplasty alone, whereas 7 cases involved stent placement after angioplasty. DCBs were initially used in 11 cases from September 2016 after conventional balloon dilatation to inhibit intimal hyperplasia. Successful recanalization was achieved in 95.5% of patients, with TICI 3 reperfusion in 18 cases and TICI 2b in 3 cases. Illustrative case is shown in Figure 1.

**Table 2:** Angiographic and procedural characteristics.

**Characteristics**	***n* = 22**
**Technical success**^**a**^	21 (95.5%)
**Modality of recanalization**
Angioplasty with DCB	11 (50.0%)
Angioplasty without DCB	3 (13.6%)
Remedial stenting	7 (31.8%)
**Postprocedural Perfusion**
TICI = 2b	3 (13.6%)
TICI = 3	18 (81.8%)
TICI = 0	1 (4.5%)
Residual stenosis^b^	4 (18.2%)
Complication rate	7 (31.8%)
Dissection	2 (9.1%)
Perforation	2 (9.1%)
Symptomatic ICH	1 (4.5%)
Asymptomatic ICH	0 (0%)
Hyperperfusion syndrome	1 (4.5%)
Branch embolization	1 (4.5%)

a *Technical success, defined as TICI ≥ 2b at the end of the intervention*.

b *Residual stenosis, defined as >50% stenosis at the end of the intervention*.

*DCB, drug-coated balloon; TICI, thrombolysis in cerebral ischemia; ICH, intracranial hemorrhage; NIHSS, National Institutes of Health Stroke Scale; SD, standard deviation; IQR, interquartile range*.

**Figure 2:**
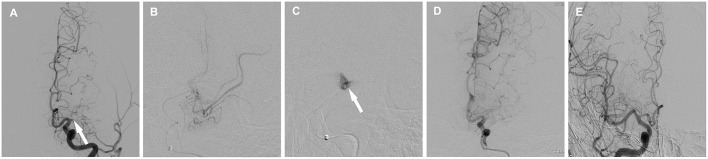
Cerebral angiographic results of patient 11 who experienced vessel perforation and coiling. **(A)** Total occlusion of the left middle cerebral artery (arrow). **(B)** Atherosclerotic and slimsy distal vasculature after selective injection. **(C)** Exudative contrast media during lesion traversal (arrow). **(D)** Angiographic result after coiling. **(E)** Angulated M1 segment of the right middle cerebral artery.

The most common intra-procedural complications were MCA perforation (two patients), with none of these patients exhibiting new clinical manifestations after coiling. Figure 2 is a representative case. Dissection during lesion traversal occurred in ~9.1% of patients (two patients), and neither limited blood flow nor otherwise resulted in persistent ischemic symptom. Post-procedural complications included hyperperfusion syndrome (symptom disappeared after strict blood pressure control and dehydration treatment), symptomatic distal embolization (patient 2), and ICH (patient 9, presented with mild headache). Thus, patient 9 was not administered aspirin and clopidogrel, underwent twist-drill drainage operation, and achieved functional independence (mRS score = 0) at discharge. The patient who suffered distal embolization presented with contralateral limb weakness and worsening of slurred speech, which was partially relieved after medical treatment and physical exercises. There were no perforating branch occlusion and death. Post-procedural improvement (NIHSS decrease of ≥2 points at discharge) occurred in 33.3% (7/21) of patients.

### Clinical and Angiographic Outcomes

Clinical and angiographic outcomes of successfully treated patients are presented in Table 3. During the first 30-day clinical follow-up, there were no recurrent cerebral ischemic events, and 80.9% (17/21) of the patients achieved a favorable clinical outcome. During the clinical follow-up period of 5.0 months, the proportion of patients with a mRS score of 0–2 was 85.7% (18/21). Only patient 9 developed ischemic stroke attributed to the cerebral ischemia of the MCA territory, but the patient refused repeat vessel imaging examination.

**Table 3:** Clinical and angiographic outcomes of successfully treated patients.

**Outcome measures**	***n* = 21**
Follow-up time, months, median (IQR)	5.0 (2.5–6.0)
30-day mRS score, median (IQR)	1.0 (0–2.0)
30-day mRS score ≤ 2	17 (80.9%)
30-day stroke or death	0 (0%)
mRS score at the last follow-up, median (IQR)	1.0 (0–2.0)
mRS score ≤ 2 at the last follow-up, median (IQR)	18 (85.7%)
mRS score ≤ 3 at the last follow-up, median (IQR)	20 (95.2%)
Ischemic event during the follow-up	1 (4.8%)
Restenosis^a^ on follow-up image, *n* (%)	0/10 (0%)
Reocclusion on follow-up image, *n* (%)	1/10 (10%)

a *Restenosis, defined as a diameter of the stenosis >50% of the target artery segment*.

*mRS, modified Rankin Scale; IQR, interquartile range*.

During the vessel imaging examination at 4.5 ± 1.84 months, cerebral angiography was obtained for nine patients. One patient was assessed *via* MR angiography. One (10%) artery was occluded again. During the endovascular procedure, this artery was first inflated with a conventional balloon, and thereafter, a Wingspan stent was deployed. Among the four patients with residual stenosis, imaging was obtained from only one patient with a patent MCA (Figure 1).

## Discussion

To the best of our knowledge, this is the largest case series of patients with non-acute symptomatic MCA occlusion treated with angioplasty or stenting. We preliminarily demonstrated the feasibility of vessel recanalization for non-acute symptomatic MCA occlusive disease that failed BMT.

In China, IAO is estimated to account for 33–50% of stroke cases and 50% of TIA cases, and is associated with a high risk of stroke recurrence and poor outcome (12). Randomized clinical trials so far have failed to show any benefit of endovascular recanalization for symptomatic IAO and favored medical therapy (13, 14). However, none of these trials have addressed the management of IAO that failed BMT. Moreover, the efficacy of extracranial–intracranial arterial bypass has yet to be conclusively demonstrated in large studies even for hemodynamic compromised patients (4). Considering that the event rate of recurrent stroke in refractory patients may be higher than what was previously reported in the randomized trial, endovascular therapy may still remain an option (15).

Revascularization of non-acute occluded MCA was previously studied in some case reports, and no agreement has been reached on the eligibility of this treatment strategy for these patients. Aghaebrahim et al. indicated that the rate of successful recanalization was 100% in four patients after PTAS, but one patient developed symptomatic ICH and only two patients achieved an mRS score of 0–2 (7). Ma et al. reported two successful stenting procedures, with one patient developing recurrent TIA and 80% restenosis (8). In our study, although only seven cases underwent stent placement after angioplasty, showing a lower percentage of stenting compared to that in previous studies (7, 16, 17), our team achieved a high rate of revascularization. Intracranial stenting could have theoretical advantages over primary angioplasty by preventing early elastic recoil, negative remodeling, and acute dissection. However, Stenting and Aggressive Medical Management for Prevention of Recurrent Stroke in Intracranial Stenosis (SAMMPRIS) investigators found that patients undergoing PTAS plus BMT for high-grade intracranial atherosclerosis have a significantly increased rate of recurrent stroke and death than patients receiving BMT alone (13). In a recent review, Padalia et al. concluded that PTAS should only be considered in symptomatic ICAS patients that are hemodynamically unstable or have repeatedly failed BMT (18). Moreover, a recent study suggested that DCB is a viable alternative to drug-eluting stents, and the new advent of Neuro Elutax DCB further increased the feasibility of using DCB in intracranial arteries (19).

Previous studies have implied that endovascular recanalization of non-acute occlusions of MCA may be safe. We reviewed seven studies involving 34 patients with occluded MCA who were treated with PTAS or angioplasty (7–10, 16, 17, 20), and 79.4% of patients achieved successful recanalization, with only two patients developing complications (7, 16). Moreover, a recent multicenter clinical trial showed a lower rate of ischemic stroke or death at 30 days when performing the endovascular recanalization at high-volume centers (> 30 intracranial stent procedures per year) (21). In our study, although seven patients (31.8%) developed periprocedural complications, only one patient who suffered distal branch occlusion was left with a persistent neurological deficit. One patient experienced hyperperfusion syndrome resulting from insufficient post-procedural blood pressure control. The case of ICH (patient 9) might be explained by cerebral hyperperfusion or hemorrhagic conversion worsened by dual antiplatelet treatment. Vessel perforation occurred in two patients, and, fortunately, there were no new symptoms and signs after coiling, implying that coiling may be an effective treatment for perforation. One case of vessel perforation occurred during lesion traversal. We suspected that the occluded M1 segment was angulated based on the symmetric characteristic of bilateral MCAs and the angulated contralateral M1 segment (Figure 2), and selective injection also confirmed the excessive tortuosity of the occluded M1 segment. A long occluded segment (>10 mm) and slimsy distal vasculature shown on micro-catheter angiography indicated that the time of actual occlusion was much earlier than that confirmed through DSA. Therefore, we extrapolated that the circuitous, angulated, and long occlusion course and long occlusion stage may be the risk factors of vessel perforation and the important causes of unsuccessful recanalization.

Despite the potential risk of the procedure, the natural course of symptomatic MCA occlusion with hemodynamic impairment is considered much poorer (1). Our series indicated that recanalization in the non-acute phase in select patients seems to be effective, with 85.7% of patients achieving a favorable outcome, and the rate of recurrent stroke is only 4.8% over a median clinical follow-up duration of 5.0 months.

However, there are several limitations of our study. First, the main limitations are its retrospective nature and monocentric design with a small sample size. Currently, there are no prospective, randomized trials that compared endovascular management with aggressive medical therapy (22). In the future, randomized controlled trials are needed to investigate whether this treatment compares favorably with the aggressive medical management in these patients. Second, in the opinion of the treating interventional physicians, patients in our study had received BMT prior to making a decision to undergo revascularization. Nevertheless, the optimal medical care likely remains less aggressive compared to the medical management performed in SAMMPRIS. Third, we obtained follow-up images only from 10 patients (47.6%), which resulted in incomplete imaging data. Fourth, the follow-up time was short; thus, the efficacy of the procedure needs to be re-evaluated in a future study with a long-term follow-up duration.

## Conclusions

This series study shows that angioplasty alone or PTAS may be feasible for the treatment of non-acute symptomatic atherosclerotic MCA occlusive disease with recurrent stroke attributed to impaired cerebral hemodynamics that failed medical treatment. It should be emphasized that revascularization of non-acute MCA occlusion is a high-risk procedure; therefore, selection of eligible patients and perfect treatment of complications are equally critical.

## Data Availability

All datasets generated for this study are included in the manuscript and/or the Supplementary Files.

## Ethics Statement

This study was carried out with the approval of the central ethics committee at Shandong Provincial Qianfoshan Hospital with written informed consent from all subjects or their legal surrogates. All subjects gave written informed consent in accordance with the Declaration of Helsinki. The protocol was approved by the central ethics committee at Shandong Provincial Qianfoshan Hospital.

## Author Contributions

JH, MZ, JiZ, and YS contributed study concepts and design. MZ, WZ, and JuZ organized the database and performed the statistical analysis. MZ, LS, HY, and WW wrote the manuscript. All authors contributed to manuscript revision and read and approved the submitted version.

### Conflict of Interest Statement

The authors declare that the research was conducted in the absence of any commercial or financial relationships that could be construed as a potential conflict of interest.
