# Supplementary material for: Endovascular Recanalization of Non-acute Symptomatic Middle Cerebral Artery Total Occlusion and Its Short-Term Outcomes
Source: Front Neurol. 2019 May 15;10:484. doi: 10.3389/fneur.2019.00484 (PMC6529837; doi:10.3389/fneur.2019.00484)
Supplement: Supplementary file 1 [file Table_1.DOCX]

Supplementary Material

# Supplementary table 1. Clinical summary of 22 patients.

| **No.** | **Sex**  **/age** | **Initial symptom** | **Symptom onset to treatment(days)** | **Occlusion confirmed to treatment(days)** | **Residualstenosis^a^(＞50%)** | **Post-op TICIgrade** | **Complication** | **Follow-up (months)** | **mRS score on the last follow-up** | **Recurrent**  **TIA or stroke** | **Restenosis^b^ or reocclusion** |
| --- | --- | --- | --- | --- | --- | --- | --- | --- | --- | --- | --- |
| 1 | M/35 | Left-side weakness | 16 | 9 | No | III | None | 6 | 0 | No | Images not available |
| 2 | M/61 | Slurred  speech | 13 | 13 | No | III | Branch  embolization | 6 | 1 | No | Reocclusion |
| 3 | M/56 | Broca aphasia, right-side weakness | 28 | 20 | Yes | IIb | None | 9 | 3 | No | Images not available |
| 4 | M/64 | Slurred  speech,  left-side weakness | 60 | 23 | No | III | None | 16 | 0 | No | Images not available |
| 5 | M/50 | Slurred  speech, left-side weakness | 15 | 7 | No | III | None | 3 | 1 | No | No |
| 6 | M/48 | Slurred  speech, right-side weakness | 11 | 11 | No | III | None | 6 | 0 | No | Images not available |
| 7 | M/49 | Slurred  speech, right-side weakness | 15 | 8 | No | III | None | 2.5 | 0 | No | No |
| 8 | M/61 | Left-side weakness | 28 | 11 | No | III | None | 6 | 3 | No | No |
| 9 | M/61 | Left-side weakness | 14 | 12 | No | III | ICH | 1.5 | 4 | Stroke | Images not available |
| 10 | M/54 | Slurred  speech, left-side weakness | 29 | 28 | Yes | III | None | 4.5 | 2 | No | No |
| 11 | M/62 | Partial broca aphasia,  cognitive impairment | 45 | 57 | NA | 0 | Perforation | 3 | 0 | No | NA |
| 12 | M/67 | Slurred  speech, central facial paralysis | 17 | 14 | Yes | IIb | HPS | 6 | 0 | No | Images not available |
| 13 | M/59 | Syncope | 45 | 16 | No | III | None | 5.5 | 0 | No | Images not available |
| 14 | M/62 | Slurred  speech, right-sided weakness | 15 | 2 | No | III | Dissection | 6 | 1 | No | Images not available |
| 15 | M/51 | Slurred  speech | 30 | 14 | No | III | None | 1.5 | 0 | No | Images not available |
| 16 | F/70 | Vertigo, right-sided weakness | 18 | 13 | No | III | None | 1.5 | 0 | No | Images not available |
| 17 | F/55 | Broca aphasia, right-side weakness | 22 | 19 | No | III | Dissection | 2.5 | 2 | No | No |
| 18 | F/62 | Slurred  speech | 14 | 11 | No | III | None | 7 | 1 | No | No |
| 19 | F/52 | Dizziness,  numbness | 18 | 15 | No | III | None | 3.5 | 0 | No | No |
| 20 | M/48 | Left-side weakness | 17 | 7 | No | III | Perforation | 7 | 2 | No | No |
| 21 | F/60 | Left-side  weakness | 40 | 0 | No | III | None | 3 | 1 | No | No |
| 22 | M/69 | Slurred  speech | 12 | 6 | Yes | IIb | None | 1 | 1 | No | Images not available |

*^a^ Residual stenosis, defined as＞50% stenosis at the end of the intervention. ^b^ Residual stenosis, defined as＞50% stenosis at the end of the intervention. mRS, modified Rankin Scale; TICI, thrombolysis in cerebral ischemia; TIA, transient ischemic stroke; ICH, intracranial hemorrhage.*
